# A comprehensive analysis of the interaction network of immunomodulatory-related differentially expressed genes, aiming to identify biomarkers associated with Parkinson’s disease

**DOI:** 10.1186/s40246-026-00929-8

**Published:** 2026-03-12

**Authors:** Jianying Gao

**Affiliations:** DEYU Healthy Management, Baoding, China

**Keywords:** Parkinson’s disease, Immunomodulatory-related differentially expressed genes, Regulatory networks, Immune infiltration, Biomarkers

## Abstract

**Background:**

Parkinson's disease (PD) is a multifactorial neurodegenerative disease that results from the interplay of genetic, environmental, and immunological factors. In order to find biomarkers linked to PD, the current study intends to thoroughly explore the interaction network of immunomodulatory-related differentially expressed genes (IMRDEGs).

**Methods:**

GeneCards database was used to obtain immunomodulation-related genes (IMRGs), and GEO database was used to obtain the expression profile dataset of GSE224913 and GSE99039 in patients with Parkinson’s disease. Using the limma package, we performed differential analysis to identify differentially expressed genes. Subsequently, we used GO, KEGG and GSEA for enrichment analysis of these differentially expressed genes. In addition, we constructed interaction networks between MRnas and transcription factors (TF), mrnas and drugs, and investigated protein–protein interaction networks (PPIs). Finally, CIBERSORT method was used for immunoinfiltration analysis to estimate the composition and abundance of immune cells.

**Results:**

A total of 6 IMRDEGs were identified in the GSE224913 and GSE99039 datasets. The main focus of enrichment analysis is on immune-related pathways, including dendritic cell development and negative regulation of leucocyte mediated immunity. Most immune cells were associated with 6 IMRDEGs; The associations were highest with neutrophils, static NK cells, and T cells with active CD4 memory. In addition, regulatory networks of mRNA-TF, mRNA-miRNA and mRNA-Drugs target genes were established.

**Conclusion:**

In summary, the six core genes (*HLA-B, HNRNPA, LILRB1, PARK7, S100A9, and SPI1*) play pivotal roles in the progression of PD. The interaction networks encompassing mRNA-TF and mRNA-drugs contribute significantly to our understanding of disease progression and the optimization of treatment strategies.

**Supplementary Information:**

The online version contains supplementary material available at 10.1186/s40246-026-00929-8.

## Introduction

Parkinson’s disease (PD) is a chronic and progressive neurodegenerative disorder primarily impacting the motor system [1, 2]. It’s characterized by the degeneration of specific neurons located in the substantia nigra region of the brain. Many motor symptoms, such as muscle stiffness, tremors, delayed movement, and trouble maintaining balance, are brought on by this deterioration [3, 4]. Even with a great deal of research, the precise etiology and pathophysiology of PD are still unclear. These factors include a complicated interaction between genetic, environmental, and immune factors. Therefore, we need to conduct data mining studies on Parkinson's disease to identify immune-related biomarkers for PD.

Recent studies has highlighted the importance of immunomodulatory processes in the etiology of Parkinson's disease (PD) [5, 6]. Inflammation and immune dysregulation have been implicated in the neurodegeneration observed in PD patients [7, 8]. Immunomodulatory-related differentially expressed genes (DEGs) represent a subset of genes that exhibit altered expression patterns in response to immune modulation, potentially as biomarkers for disease progression and response to therapy.

This study aims to conduct a comprehensive analysis of the IMRDEGs interaction network. By employing bioinformatics tools and techniques, we will investigate the intricate relationships among these genes, seeking to identify key players and regulatory hubs within the network. Our objective is to find novel biomarkers linked to PD because doing so may help us create more efficient diagnostic, prognostic, and therapeutic approaches.

By understanding the immunomodulatory gene interactions in PD, we hope to gain insights into the underlying mechanisms of neurodegeneration and immune dysregulation in this disease. Our objective is to identify new biomarkers linked to PD that may facilitate the creation of more robust therapeutic, diagnostic, and prognostic approaches.

## Materials and methods

### Data download

The R package GEOquery2.70.0 [9] was utilized to retrieve the expression profiling datasets GSE22491 and GSE99039 concerning PD patients from the GEO database [10]. Both GSE22491 and GSE99039 were sourced from Homo sapiens. There were 18 samples in GSE22491, comprising 10 PBMC samples from PD patients and 8 matched samples from healthy controls. Using these datasets, peripheral blood mononuclear cells (PBMC) from both healthy people and patients with Parkinson's disease were subjected to expression profiling. The data platform utilized was the probe version GPL6480 Agilent-014850 Whole Human Genome Microarray 4 × 44 K G4112F. The GSE99039 dataset comprised 438 whole blood samples that were used for expression profiling. They did not include whole blood samples from 48 patients with other neurodegenerative diseases. The samples were from patients who had idiopathic Parkinson's disease (IPD), patients who had other neurodegenerative disorders (NDD), and healthy individuals. The study included 205 whole blood samples from healthy controls and 48 whole blood samples from patients with different neurodegenerative disorders. Furthermore, 233 whole blood samples from patients with a definite diagnosis of IPD were taken. GPL570 [HG-U133_Plus_2] was the data platform where the dataset was hosted. The U133 Plus 2.0 Array The Affymetrix Human Genome System.

The datasets’ probe name annotations were derived from the chip GPL platform files. The PBMC samples from dataset GSE22491 had the Expression profiling data of 10 PD patients, while the matched 8 healthy control samples also included some expression profiling data. Additionally, 233 whole blood samples from IPD patients and 205 matched healthy control samples’ Expression profiling data were included in dataset GSE99039.

The treatment of diseases requires immunomodulation. This is why a search of the GeneCards database (https://www.genecards.org/) turned up immunomodulatory-related genes (IMRGs) /) [11]. A wealth of information on human genes is available on GeneCards. By using the search term “Immunomodulatory,” we limited the selection of “Protein Coding” genes that had a “Relevance score > 0.500.” There were 505 IMRGs in all.

### Parkinson’s disease-related differentially expressed genes

We first used the limma package(3.58.1) to normalize the datasets GSE22491 and GSE99039 in order to discover the potential mechanistic functions, associated biological characteristics, and pathways of differentially expressed genes (DEGs) in PD [12]. After that, differential analysis performed to the datasets revealed genes that were differentially expressed (DEGs) between the diseased group and the control group. DEGs for additional research were chosen based on their combination of |logFC|> 0 and P. adj. < 0.05. It was determined that genes with logFC > 0 and P. adj. < 0.05 were upregulated, while genes with logFC < 0 and P. adj. < 0.05 were considered downregulated.

The common differentially expressed genes (Co-DEGs) were first found by creating a Venn diagram, which allowed us to discover the immunomodulatory-related differentially expressed genes (IMRDEGs) associated with Parkinson’s disease. This diagram was created by intersecting all DEGs from the GSE22491 and GSE99039 datasets, with a logFC value greater than 0 and a significance level of 0.05. Subsequently, we intersected the Co-DEGs with the IMRGs and plotted another Venn diagram. The differential analysis results were displayed using volcano plots created by the R package ggplot2(3.4.4)and heat maps created by the R package heat map.

### Functional enrichment analysis (GO) and pathway enrichment (KEGG) analysis for differentially expressed genes

Using gene ontology (GO) analysis is a commonly utilized technique for conducting comprehensive functional enrichment studies covering molecular functions (MF), cellular components (CC), and biological processes (BP). GO annotations on IMRDEGs were analyzed using the R package clusterProfiler(4.10.0). In order to identify statistically significant terms with P. adj. < 0.05 and an FDR value (q. value) < 0.05, the significant term selection criteria were developed. The Benjamini-Hochberg (BH) approach of P-value correction was used.

### GSEA

To establish a gene set's contribution to a phenotype, Gene Set Enrichment Analysis (GSEA) [13] is used to assess the distribution trend of the gene set within a gene list sorted by phenotypic relevance. The genes from the GSE22491 and GSE99039 datasets were initially categorized into categories in this analysis according to positive and negative logFC values. Then, for each gene that displayed differential expression in the positive and negative logFC groups, we ran an enrichment analysis using the clusterProfiler program. The following parameters were used in this GSEA: We'll perform 100,000 permutations, with the seed set to 2023. Each gene set will contain at least 10 genes and up to 500 genes. P-value correction will be performed using the Benjamini-Hochberg (BH) method. The Molecular Signatures Database (MSigDB) provided the gene set c2.all.v2022.1.Hs.symbols.gmt. We used an FDR value (q. value) < 0.25 and P. adj. < 0.05 as criteria to find highly enriched gene sets.

### Protein–protein interaction (PPI) network

Proteins engage in mutual interactions to form the PPI network, which is crucial for biological functions such as transmission of signals, metabolism, gene regulation, and cell cycle regulation. Understanding the intricate relationships between proteins in biological systems is essential for unraveling the inner workings of these systems. It allows us to understand how proteins respond to biological signals and energy substances, especially in unique physiological conditions like diseases. Additionally, it helps us uncover the functional connections that exist among proteins. STRING is a highly useful database for analyzing protein–protein interactions, providing a comprehensive collection of known and predicted interactions.

The PPI network for the selected IMRDEGs in this study was built using the STRING database. To guarantee the dependability of the interactions, we employed a low confidence minimum necessary interaction score of 0.150. Following that, Cytoscape (version 3.9.1) was used to show the PPI network model [14], and it was determined that the IMRDEGs were hub genes critical to Parkinson's disease.

### Construction of mRNA-TF and mRNA-drugs interaction networks

The CHIPBase2.0 database (third iteration) predicts a vast number of transcriptional regulatory links between transcription factors (TFs) and genes, amounting to millions [15]. It utilizes Chip-seq data of DNA-binding proteins to identify many binding motifs. The hTFtarget database thoroughly collects information on human transcription factors and the regulatory targets that correspond with them. This study employed the CHIPBase database (version 3.0) and the HTF target database to identify the transcription factors (TFs). The Cytoscape software was utilized to demonstrate the interactions among the transcription factors (TFs) that bind to the hub genes.

In addition, the Comparative Toxicogenomics Database (CTD) was employed to predict potential pharmaceuticals or small-molecule medications that exhibit interactions with hub genes [16]. Subsequently, the Cytoscape software was employed to visually represent the interconnections of mRNA-TF and mRNA-Drugs networks.

### Immune infiltration analysis (CIBERSORT)

The immune infiltration analysis algorithm CIBERSORT [17] employs the concept of linear support vector regression to deconvolut transcriptome expression matrices. This algorithm is utilized to estimate the quantity and composition of immune cells among mixed-cell populations. The matrix data from the Parkinson's disease (PD) dataset, comprising both the disease and control groups, was subjected to analysis using the CIBERSORT program [18]. By combining the LM22 signature gene matrix and excluding data with immune cell enrichment scores higher than zero, we were able to measure and document the exact amounts of immune cell infiltration. The Spearman algorithm was utilized to ascertain the correlation between the different immune cells in the PD dataset samples, and the results were displayed using the R package ggplot2. Then, a correlation scatter plot was created by combining the gene expression matrix of the Parkinson's disease dataset using the R program ggplot2. This was carried out to see whether immune cells and hub genes are related.

### Statistical analysis

All of our data processing and analysis is based on R software (Version 4.1.2). Continuous variables are presented in the form of mean ± standard deviation. The comparison between the two groups was performed using the Wilcoxon rank sum test (Wilcoxon rank sum Test) to assess statistically significant differences on different measures between patients with Parkinson’s disease (PD) and healthy controls. In addition, correlation analysis involving different molecules in the study was calculated using spearman correlation analysis. In order to determine the relationship between genes, proteins and immune cell abundance, correlation coefficients were calculated by this method.

In the part of immune infiltration analysis, CIBERSORT method was used to deconvolution the matrix data of the dataset, and the data with immune cell enrichment score greater than zero was screened by combining with LM22 characteristic gene matrix, and the specific results of immune cell infiltration abundance matrix were finally displayed. The correlation between immune cells and key genes was comprehensively analyzed, and the correlation point map was drawn by R package ggplot2 to show the research results. All statistical results were judged with a P value less than 0.05 as a significant difference to ensure the reliability and scientificity of the results.

## Results

**Fig. 1:**
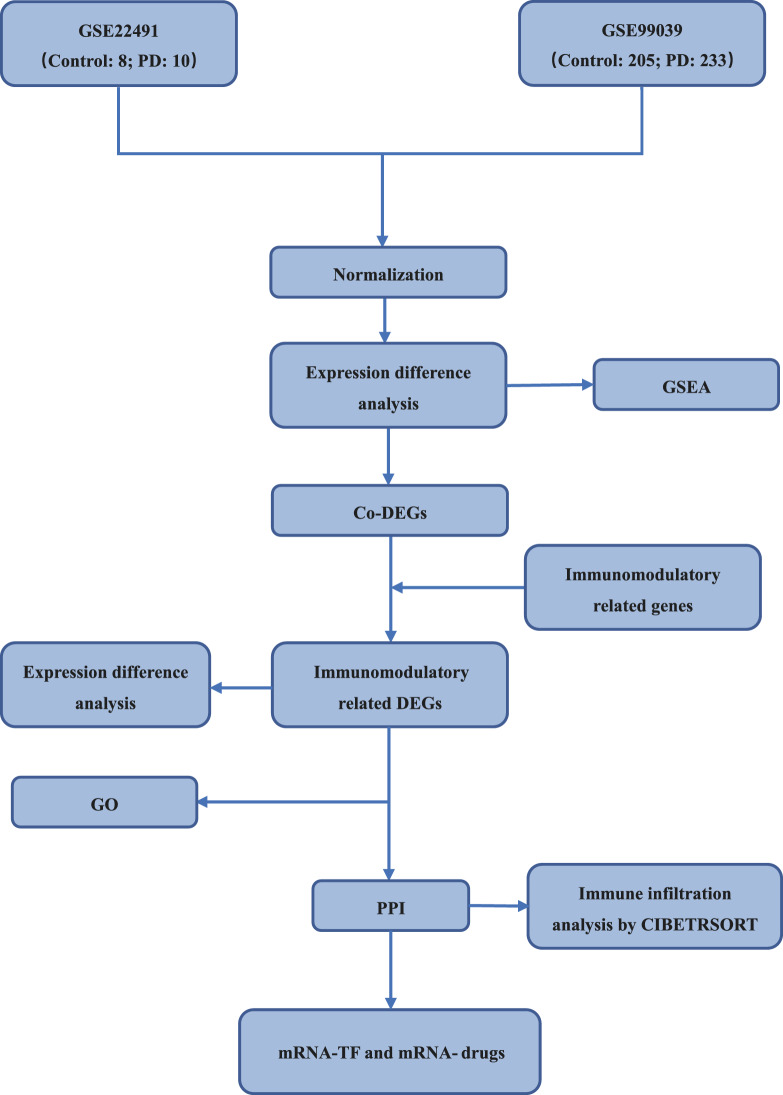
Technology roadmap

The Workflow Diagram of this study is shown in Fig. 1.

### Standardized processing of Parkinson’s disease datasets

**Fig. 2:**
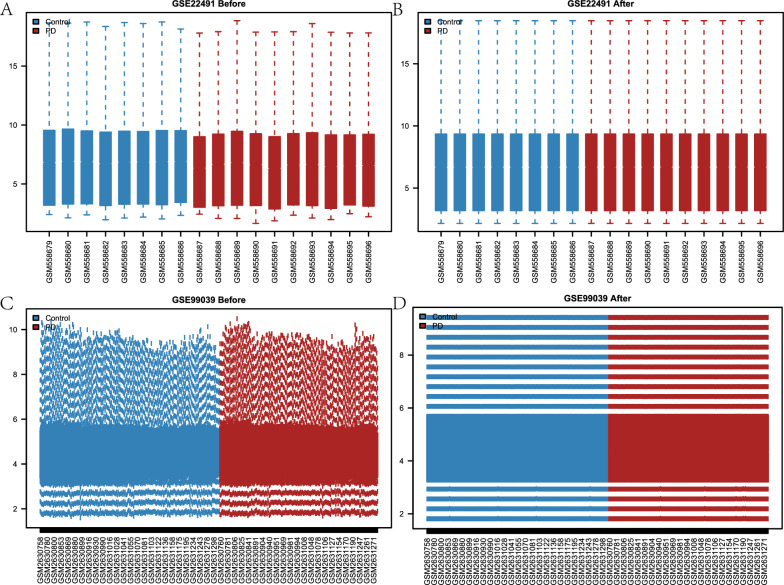
Standardization of Parkinson's disease (PD) data sets. **A**, **B** boxplot plot of GSE22491 data set before normalization (**A**) and boxplot plot of GSE22491 data set after normalization (**B**). **C**, **D** boxplot plot of GSE99039 data set before normalization (**C**) and boxplot plot of GSE22491 data set after normalization (**D**)

First, the R utility limma was used to perform standardized processing on the two PD datasets, GSE22491 and GSE99039 (Fig. 2A–D). There were 18 samples in the GSE22491 dataset: 10 disease samples and 8 control samples (Fig. 2A, B) (Table 1). The GSE99039 dataset contained 438 samples: 205 control and 233 disease samples (Fig. 2C, D). After standardization of processing, as shown in Fig. 2A–D, the PD datasets GSE22491 and GSE99039 expression profile data showed a consistent expression pattern across samples, with the majority of the batch effects within samples eliminated.

**Table 1:** List of Parkinson’s disease datasets information

	GSE22491	GSE99039
Platform	GPL6480	GPL570
Type	Expression profiling by array	Expression profiling by array
Species	Homo sapiens	Homo sapiens
Tissue	PBMC	Whole blood
Samples in PD group	10	233
Samples in Control group	8	205
Reference	Transcriptional profile of Parkinson blood mononuclear cells with LRRK2 mutation	Analysis of blood-based gene expression in idiopathic Parkinson disease

PBMC: Peripheral mononuclear blood cells; PD: Parkinson’s disease

### Analysis of Parkinson’s disease-related differential genes

**Fig. 3:**
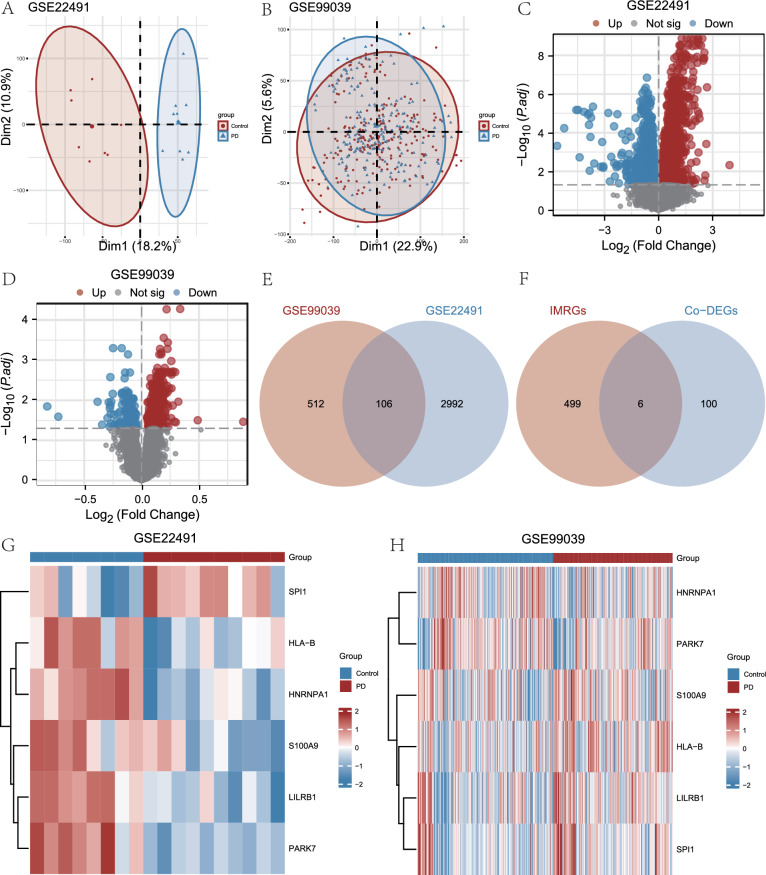
Genes with differential Expression in Parkinson's disease. **A**, **B** PCA of the GSE99039 dataset (**B**) with the GSE22491 dataset (**A**). **C** The volcano plot visually represents the variations in gene expression levels observed between the disease group (PD) and the control group within the GSE22491 dataset **D** The GSE99039 dataset includes a volcano map that visually represents the differences in gene expression between the control group, referred to as PD, and the disease group, referred to as disease **E** The Venn diagram illustrates the differential expression of genes between the GSE99039 and GSE22491 datasets. **F** Venn diagram showing the IMRGs and Co-DEGs in the datasets. **G**, **H** Intricate heatmaps of IMRDEGs from the GSE99039 (**H**) and GSE22491 (**G**) datasets. Principal component analysis (PCA), common differentially expressed genes (Co-DEGs), immunomodulatory related genes (IMRGs), and Parkinson's disease (PD)

When the expression profile data from the GSE99039 and GSE22491 datasets were first collected, principal component analysis (PCA) was applied. The PD group and the control group were created using these datasets. Feature vectors are extracted from high-dimensional data using PCA, a data dimension reduction technique, and then these features are shown in two or three dimensions. Subsequently, we presented the PCA results for the GSE22491 dataset (Fig. 3A) and the GSE99039 dataset (Fig. 3B).

Using the limma program, we normalized the GSE22491 and GSE99039 datasets for the PD and Control groups. In order to find differentially expressed genes (DEGs) among various groups within the two datasets about Parkinson's disease, differential analysis was performed on the GSE22491 and GSE99039 datasets using the limma software. This enabled us to examine the disparities in gene expression between the Parkinson’s disease cohort and the Control cohort in PD.

The results are as follows: The GSE22491 dataset yielded 19,595 DEGs, with 3098 genes satisfying the threshold of |logFC|> 0 and P. adj. < 0.05. Below the established threshold, a total of 1579 genes had positive logFC and showed elevated expression levels in the disease group relative to the control group, suggesting upregulation. In contrast, 1519 genes demonstrated downregulation. To display the differential analysis findings for this dataset, we created a volcano plot (Fig. 3C). Of the 21,655 DEGs found in the GSE99039 dataset, 618 genes satisfied the requirements of P. adj. < 0.05 and |logFC|> 0. Among these, 437 genes had positive logFC (upregulated genes), and 181 had negative logFC (downregulated genes). The differential analysis results for this dataset were also plotted as a volcano plot (Fig. 3D).

To find IMRDEGs, we first intersected the DEG sets from the GSE22491 and GSE99039 datasets with |logFC|> 0 and P. adj. < 0.05. This yielded 106 Co-DEGs specific to PD, which we visualized using a Venn diagram (Fig. 3E). Subsequently, we intersected the Co-DEG set with a list of immunomodulatory-related genes (IMRGs) to identify six IMRDEGs specific to Parkinson’s disease. These six IMRDEGs were: *HLA-B, HNRNPA, LILRB1, PARK7*, *S100A9*, and SPI1. We plotted a Venn diagram to illustrate this intersection (Fig. 3F).

We next looked at the differences in these six IMRDEGs' expression patterns between the control group and the disease group after analyzing the Venn diagram data. This analysis used the GSE22491 dataset (Fig. 3G) and the GSE99039 dataset (Fig. 3H). The differential analysis results for these six IMRDEGs were visualized as heatmaps using the R package heatmap (Fig. 3G, H).

### Correlation analysis of IMRDEGs in Parkinson’s disease datasets

**Fig. 4:**
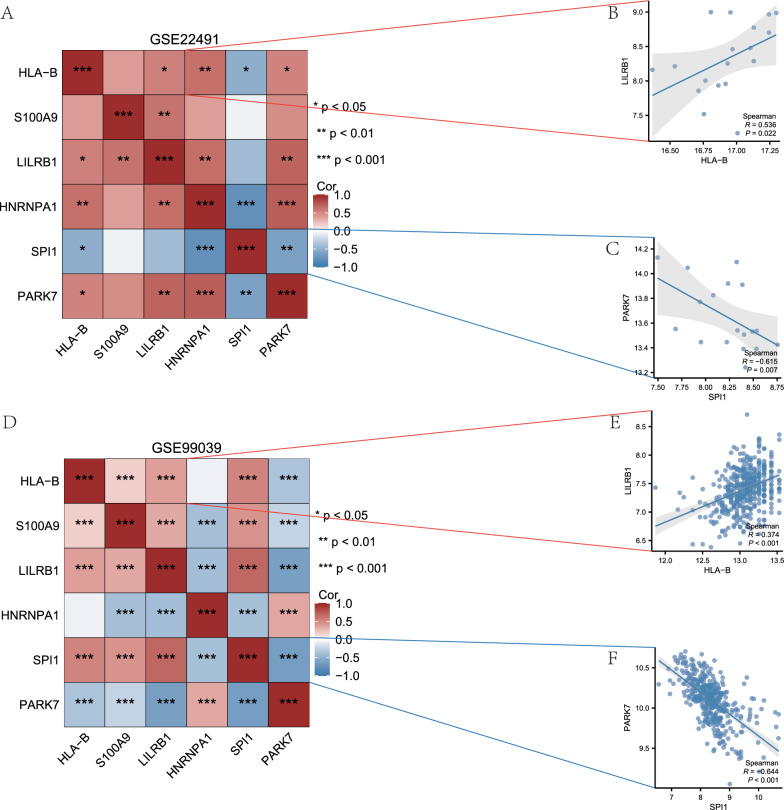
Correlation Analysis of IMRDEGs in PD Datasets (PD). **A** Correlation heatmap results of IMRDEGs in the GSE22491 dataset. **B**, **C** Correlation scatter plot results of IMRDEGs *HLA-B* and *LILRB1* (**B**), SPI1, and PARK7 (**C**). **D** Correlation heatmap results of IMRDEGs in the GSE99039 dataset. **E**, **F** Correlation scatter plot results of IMRDEGs *HLA-B* and *PLAC8* (**E**), *SPI1*, and *PARK7* (**F**). In a correlation scatter plot, an absolute correlation coefficient exceeding 0.8 signifies a strong correlation. A value ranging from 0.5 to 0.8 indicates a moderate correlation, while a value between 0.3 and 0.5 indicates a weak correlation. A value below 0.3 indicates a weak or nonexistent correlation

In order to investigate the correlation between the six IMRDEGs in the GSE22491 and GSE99039 datasets (Table 2), in the GSE22491 dataset, we used the Spearman method to look at the expression levels of these six IMRDEGs. The correlation heatmap was used to display the results (Fig. 4A). Based on the results, a significant statistical relationship (P < 0.05) was seen in the majority of the correlations among the six IMRDEGs within the GSE22491 dataset. Following that, we utilized the six IMRDEG expression levels from the GSE99039 dataset. These levels were subjected to analysis utilizing the Spearman algorithm, and the outcomes were shown in the form of a correlation heatmap (Fig. 4D). The findings demonstrated statistical significance (P < 0.05) in the relationships among most of the six IMRDEGs in the GSE99039 dataset.

**Table 2:** Immunomodulatory related genes related genes list

Immunomodulatory related genes					
PIBF1	IL1RN	IL17RB	CX3CL1	CCL7	AFP
TNF	CAMP	LST1	CUL4B	NAGLU	F2R
IFNG	CCL5	CD55	SELE	PDE7A	PTGS1
IL10	LTA	BCL2L1	C4BPA	HLA-DPA1	ERCC1
IFNA1	IL1RAPL2	C1QA	IL37	MEIS2	TRAF1
IL2	PTGS2	FOXP3	PTPRC	S100A6	CPQ
IL6	PAEP	HRH4	FCGR1A	CD300A	ORM2
ITFG1	CCL2	GAA	ANGPT1	CXCL13	CSF1R
IL1B	IKZF1	CYCS	SLC29A3	TMEM63A	EGF
IFNA2	IL17A	SCGB1A1	NAIP	BST2	FKBP5
CRBN	IL15	DPEP1	SIAE	CCL8	EIF2AK3
CXCL8	TLR2	IRF4	ABHD16A	NIPSNAP1	IL2RG
IFNB1	EPO	IL23A	WFDC1	NIPSNAP2	P2RY12
IFNL3	LBR	COMP	FOS	RARRES2	TRPA1
IL2RA	CTLA4	IL11	GAPDH	C1QTNF3	PARK7
IFNL1	SEMA7A	MYC	NLRP3	CLEC16A	IL1RAP
IL4	CSF3	ENG	SEMA4D	OXT	RIPK2
IFNL2	CD274	HLA-DQB1	TNFAIP3	GDF7	CD34
IL5	MIF	VCAM1	FLNB	IFNA5	CRAT
CSF2	STAT1	HP	CD163	BPIFA1	METAP2
IL16	CD40LG	BAX	DOCK2	IFNA6	NPR3
IL2RB	BMP6	HLA-A	EGR2	CORT	ATP6V0A2
CST9	TLR9	IL22	CCL20	IFNA14	FOSL1
TLR4	CCL4	IGHE	UMOD	IFNA21	TNFRSF9
IDO1	HGF	PRDM1	CD226	IFNA8	CAMK1
ICAM1	IL1R1	HLA-DQA1	IDO2	UCN	CFP
IL1A	SLURP1	IL18R1	FGF2	IFNA13	MAPRE2
IL13	HMOX1	PCNA	CAT	IFNA4	UBC
IL18	PDE4A	CYP19A1	SRC	SIGLEC14	NFKBIB
VEGFA	VDR	RORC	WNT5A	IFNA10	ATP5PO
ITGB2	B2M	S100A9	CASP7	IFNA17	JUNB
HLA-G	IL12B	SIGLEC5	HSPA1A	IFNA7	PDE1A
TGFB1	S100A7	CSN2	NPPA	IFNA16	SORBS2
LGALS1	IL1F10	THBS1	BSG	LILRA3	FOLR3
ACKR2	NOS2	IRF1	MLXIPL	AHR	RAB27B
CEBPD	HLA-DRB1	SST	BMI1	GHRL	SCARF1
CERK	CXCR4	EIF2S1	GDF15	MMP1	BTN2A2
GFI1B	CSF1	VTCN1	PLP1	ORM1	CCL16
SPIB	JUN	CLEC12A	IRF2	MAP2K1	CDCA5
CD53	STAT3	IL17B	SIGLEC7	EIF4E	SCGB3A2
SH2D1B	TLR7	IFNAR2	TNFRSF6B	GATA2	CCPG1
TSPAN32	PLA2G3	IGF1	CCL17	CD46	MRPL28
CHUK	CLEC4E	P2RY11	IL15RA	HNRNPA1	NANOG
FCGR2B	TMPO	SPHK2	MICB	ITGAV	CCL15
HDAC9	IL3	CEACAM6	MICA	APAF1	CEACAM4
FCGR2A	TNFRSF1A	SERPINB1	SIGLEC9	BMP2	KRT76
STAT4	FASLG	HSPA1B	NFKBIA	SPHK1	CLEC4C
RBX1	CD40	MAPK14	SELL	ATG5	IGHG1
CYP3A4	CD80	NR1H2	TERT	BECN1	BATF2
FGFR2	LY9	CD200R1	TFRC	NME1	CDK6
CCND1	TLR3	TNFRSF1B	G6PD	PTH	ITGB1
KDR	PPARG	CD14	TF	TNFSF13B	APC
CFH	MAPK1	NT5E	EPOR	CD151	CAV1
TIMP1	MBP	APOE	EDN1	CD47	CYP1A1
VIP	BIRC5	NR3C1	LYN	CLCN1	VWF
DNMT1	HMGCR	CD28	CD8A	CRHR1	WT1
MMP3	CNR2	CD79A	SLC16A1	CST3	C5
TLR1	CD4	ITGAM	XDH	ATP6V0A1	ITGAX
FCRL6	LGALS3	SIK2	SOCS1	CD63	THBD
CTNNB1	CD86	F2RL1	TBX21	CEACAM5	CHRNA7
MX1	DDB1	MBL2	CEBPB	CRHR2	ITGB7
CYP1A2	BIRC3	CCL11	HTR2B	F11R	TLR6
ITGAL	ITGA4	GAL	NPPB	FBXO7	TNFRSF10A
PIM1	CRP	IRF3	OPRD1	GH1	APOH
PRKCQ	LTF	LILRB1	CYP7A1	JAG2	CYP3A5
BRD4	DHODH	TGFB2	MCAM	KLF1	DKK1
AGER	CD200	EIF2AK2	LGALS3BP	PODXL	VTN
FOLH1	TNFRSF11B	SPP1	PIGR	PSMD4	CD84
HSP90B1	CXCR3	IL12A	IL22RA2	SDC1	KLRC1
NGFR	CASP3	CD209	SIGLEC10	SPI1	KLRD1
CD38	IKZF3	CRH	SIGLEC6	RPS27A	GSTT1
GATA1	CUL4A	CXCL9	IL33	S100A8	CCR5
RANBP2	TNFSF11	IL27	LILRA1	SFTPC	POMC
CTSS	HLA-B	FCGR3B	SIGLEC11	SRSF1	ADCYAP1
EIF2AK4	CXCL10	BRAF	TNFSF9	TNFRSF18	CYP27B1
HLA-DPB1	IL7	JAK1	PDCD1	CD33	BIRC2
HNRNPA2B1	LGALS9	MPO	CNR1	IL21	KLK3
TNFSF13	MAPK3	SOD1	CCL3	CIITA	MOG
CD9	ISG15	APP	TLR8	CXCL2	TP53
EOMES	CD276	ALB	ADIPOQ	LEP	FAS
HSPA1L	JAK2	STAT5A	ADORA2A	NFKB1	CD69
IL1RAPL1	TNFSF10	CLEC7A	XIAP	HLA-E	ADAM17
GATA3	INS	CX3CR1	ANXA5	ELANE	IL6R
FCGR3A	LBP	LILRB2	CXCL12	CSNK1A1	MADCAM1
CCR1

Furthermore, we presented the correlation analysis results between genes with similar correlation trends in the correlation heatmaps through scatter plots (Fig. 4B, C, E, F). Among the six IMRDEGs, *HLA-B* and *LILRB1* (r = 0.536, P = 0.022, Fig. 4B) exhibited a moderate correlation in their expression levels in the GSE22491 dataset. In contrast, *PARK7* and *SPI1* (r = − 0.615, P = 0.007, Fig. 4C) showed a moderate negative correlation in their expression levels in the same dataset. In contrast, *HLA-B* and *LILRB1* (r = 0.374, P < 0.001, Fig. 4E) displayed a weak correlation in their expression levels in the GSE99039 dataset, while *SPI1* and *PARK7* (r = − 0.644, P < 0.001, Fig. 4F) showed a moderately negative association in the dataset for their expression levels.

### Analysis of expression differences in IMRDEGs

In order to get additional insight into the differences in the expression of the six IMRDEGs, a comprehensive analysis was carried out to look at the relationship between these genes' expression levels in the GSE22491 and GSE99039 datasets and their importance regarding illness state, relative to the control group.

**Fig. 5:**
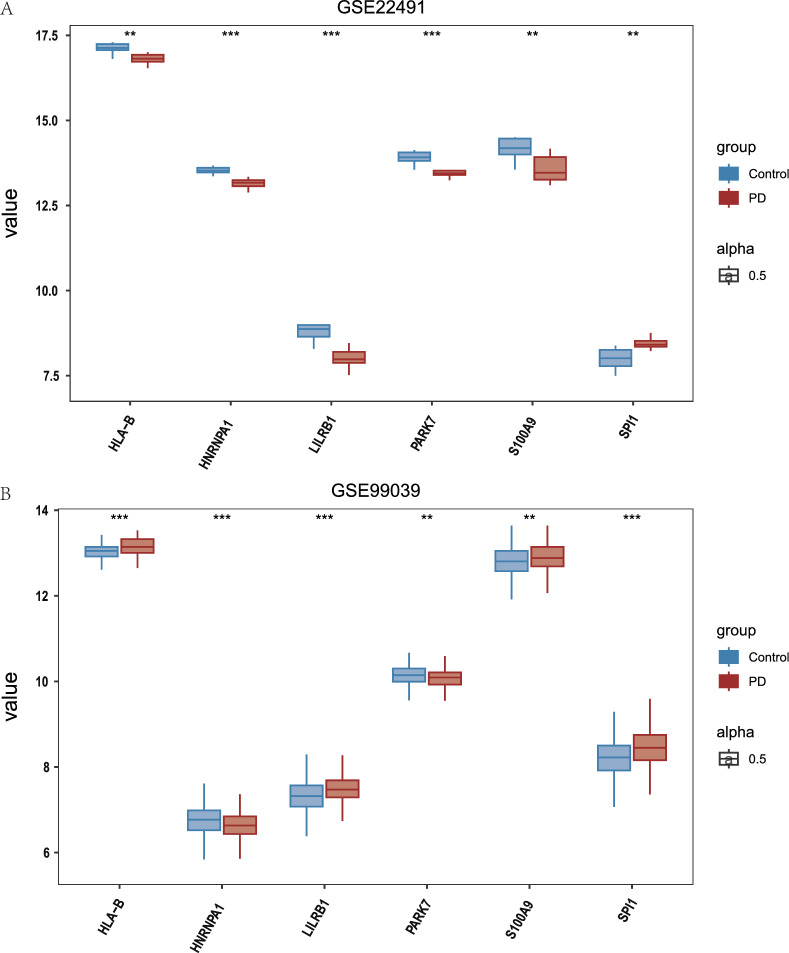
Analysis of Expression Differences in IMRDEGs. **A** Expression difference analysis group comparison chart of IMRDEGs in the GSE22491 dataset. **B** Group comparison chart of expression difference analysis of IMRDEGs in the GSE99039 dataset. The following symbols denote different levels of statistical significance: “**” denotes P < 0.01, indicates high statistical significance, and “***” denotes P <  0.001, indicates extremely high statistical significance

The present investigation aimed to examine the differences in the expression levels of the six IMRDEGs present in the GSE22491 dataset. Specifically, we employed the Wilcoxon signed rank test to compare the disease group with the control group. The GSE22491 dataset's results showed that there were statistically significant differences in the expression of all six IMRDEGs between the PD and control groups (Fig. 5A): *HLA-B* (marked with ** indicating P < 0.01), *HNRNPA* (marked with *** indicating P < 0.001), *LILRB1* (marked with *** indicating P < 0.001), *PARK7* (marked with *** indicating P < 0.001), *S100A9* (marked with ** indicating P < 0.01), and *SPI1* (marked with ** indicating P < 0.01).

Subsequently, we performed similar studies to explore the fluctuations in the Expression of the six IMRDEGs inside the GSE99039 dataset. We examined the GSE99039 dataset’s gene expression levels, comparing the PD and control groups. The signed-rank Wilcoxon test was used for this study. In the GSE99039 dataset, the findings showed that there were statistically significant variations in the expression of all six IMRDEGs between the PD and control groups (Fig. 5B): *HLA-B* (marked with *** indicating P < 0.001), *HNRNPA* (marked with *** indicating P < 0.001), *LILRB1* (marked with *** indicating P < 0.001), *PARK7* (marked with ** indicating P < 0.01), *S100A9* (marked with ** indicating P < 0.01), and *SPI1* (marked with *** indicating P < 0.001).

### GO and KEGG of IMRDEGs

**Fig. 6:**
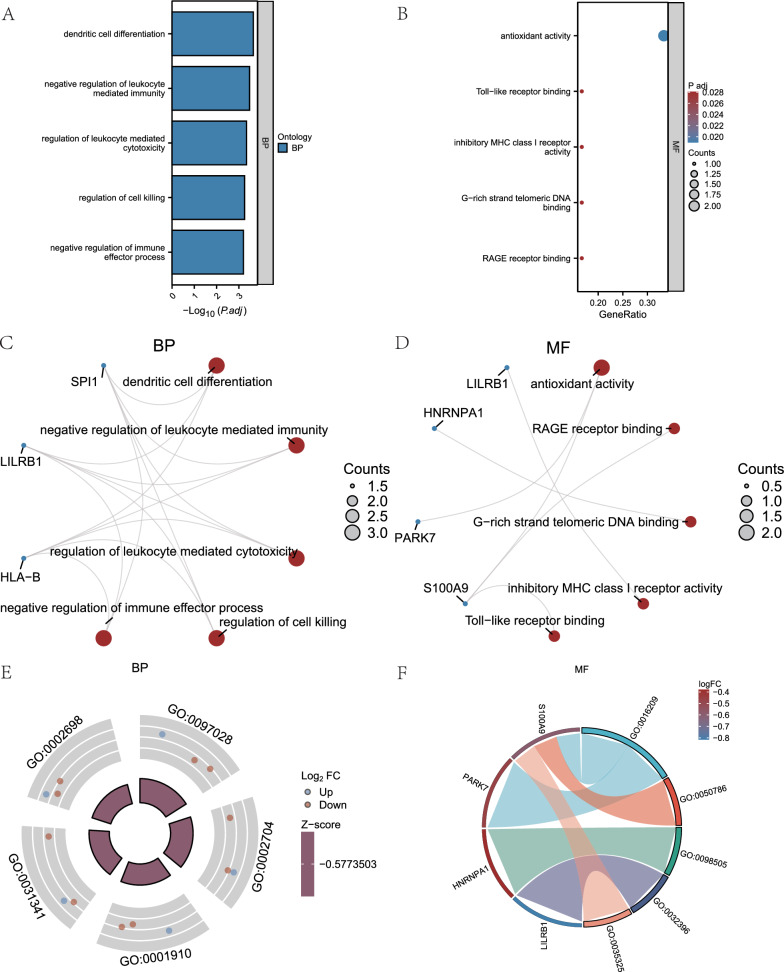
GO Enrichment Analysis of IMRDEGs. **A**, **B** The GO functional enrichment analysis of IMRDEGs is presented as a bar chart for the BP results (**A**) and a bubble chart for the CC results (**B**). **C**, **D** GO functional enrichment study of IMRDEGs circular network diagrams showing MF pathways (**D**) and BP pathways (**C**). **E**, **F**. Circle plot (**E**) and chord diagram (**F**) presentations of the results of the GO functional enrichment study combining logFC of IMRDEGs in GSE22491. The P. adj. value of the pathway is indicated by the bubble color in the bubble chart (**A**), while the y-axis displays GO words. Certain genes are represented by blue dots in the network diagrams (**B**–**D**, **F**), while specific pathways are represented by red circles. IMRDEGs: Differentially expressed genes associated with immunomodulation. For GO and KEGG enrichment items, P. adj < 0.05 and FDR value (q.value) < 0.05 are the selection criteria

Our objective was to investigate the relationships between various biological pathways, cellular constituents, molecular activities, and processes that are associated to Parkinson's disease (PD). First, for each of the six IMRDEGs, we ran a GO functional enrichment analysis (Table 3). The criterion for selecting upgraded items included a statistical significance level of P. adj. < 0.05 and an FDR value (q.value) < 0.05. According to the study's results, six immune modulatory receptor-related genes (IMRDEGs) were found to be primarily enriched in the blood plasma (BP) of Parkinson's disease patients. These genes include those that regulate leukocyte-mediated immunity, leukocyte-mediated cytotoxicity, cell death, and negative control of immune effector activities. They also regulate dendritic cell development. Furthermore, they exhibited MF enrichment in terms of antioxidant activity, binding of the rage receptor, binding of the G-rich strand telomeric DNA, binding of the inhibitory MHC class I receptor, and binding of the Toll-like receptor. Figure 6A shows the bar chart used to display the BP results of the GO functional enrichment study, whereas the bubble chart was used to display the CC results (Fig. 6B). Furthermore, we have provided network diagrams that show the BP and MF pathways obtained from the functional enrichment analysis of the GO gene (Fig. 6C, D).

**Table 3:** GO enrichment analysis results of Immunomodulatory related differentially expressed genes

Ontology	ID	Description	GeneRatio	BgRatio	pvalue	p.adjust
BP	GO:0097028	Dendritic cell differentiation	3/6	47/18800	2.91e-07	0.0002
BP	GO:0002704	Negative regulation of leukocyte mediated immunity	3/6	67/18800	8.59e-07	0.0003
BP	GO:0001910	Regulation of leukocyte mediated cytotoxicity	3/6	85/18800	1.77e-06	0.0005
BP	GO:0031341	Regulation of cell killing	3/6	100/18800	2.89e-06	0.0006
BP	GO:0002698	Negative regulation of immune effector process	3/6	112/18800	4.06e-06	0.0006
MF	GO:0016209	Antioxidant activity	2/6	85/18410	0.0003	0.0190
MF	GO:0050786	RAGE receptor binding	1/6	10/18410	0.0033	0.0281
MF	GO:0098505	G-rich strand telomeric DNA binding	1/6	10/18410	0.0033	0.0281
MF	GO:0032396	Inhibitory MHC class I receptor activity	1/6	12/18410	0.0039	0.0281
MF	GO:0035325	Toll-like receptor binding	1/6	12/18410	0.0039	0.0281

GO: Gene Ontology; BP: Biological process; MF: Molecular function。

A thorough logFC GO functional enrichment analysis was performed on the six IMRDEGs. The z-score for each gene in GSE22491 was determined using the logFC values derived from the differential analysis results of the six IMRDEGs. GO functional enrichment analysis was employed in this work. A circular plot was used to display the results of the enrichment study for the GSE22491 CC route when paired with logFC (Fig. 6E). In contrast, the enrichment analysis results of the MF pathway were presented using a chord diagram (Fig. 6F).

### GSEA of PD datasets

**Fig. 7:**
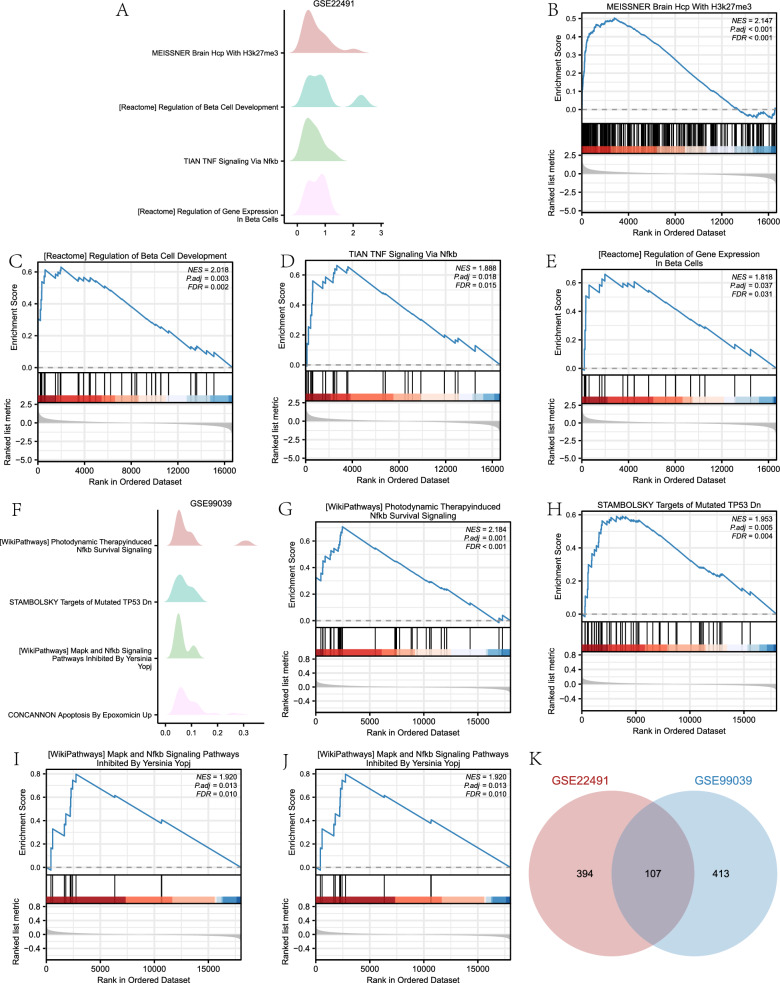
GSEA Enrichment Analysis of PD Datasets GSE22491 and GSE99039. **A** The key four biological characteristics of the GSE22491 dataset as analyzed using GSEA enrichment. Significant enrichment of differentially expressed genes in the brain hcp with h3k27me3 (**B**), regulation of beta cell development (**C**), TNF signaling via nfkb (**D**), and regulation of gene expression in beta cell pathways (**E**) pathways was found in the GSE22491 dataset. **F** The primary four biological characteristics identified by the GSEA analysis of the GSE99039 dataset. **G**–**J** Significantly, the GSE99039 dataset exhibited elevated levels of gene enrichment in relation to the survival signaling pathways induced by photodynamic therapy (**G**), mutant tp53 dn targets (**H**), yersinia you-inhibited mark and nfkb signaling pathways (**I**), and epoxomicin-induced apoptosis (**J**). K. Venn diagram showing pathways that are considerably enriched based on the GSEA enrichment analysis results of the GSE99039 and GSE22491 psoriasis datasets. GSEA is an abbreviation for Gene Set Enrichment Analysis. The criterion for significant enrichment in GSEA analysis are P. adj. < 0.05 and FDR value (q.value) < 0.25. GSEA is a gene set enrichment analysis; PD stands for Parkinson’s disease

The Gene Set Enrichment Analysis (GSEA) was employed in the GSE22491 and GSE99039 datasets to examine the associations among gene expression, biological processes, cellular components, and molecular functions. The objective was to evaluate the impact of gene expression levels on the occurrence of Parkinson’s disease (PD). An FDR value (q.value) < 0.25 and a P. adj. < 0.05 indicated significant enrichment. As shown in Table 4 and Fig. 7a–e, the differentially expressed genes in the GSE22491 dataset were significantly enriched in pathways including regulation of beta cell development, brain hcp via nfkb, brain hcp with h3k27me3, and regulation of gene expression in beta cells. In contrast, the differentially expressed genes in the gse99039 dataset exhibited significant enrichment in various pathways, including those involved in the induction of nfkb survival signaling (as shown in Fig. 7g), targets of mutated tp53 dn (as shown in Fig. 7h), apoptosis induced by epoxomicin up (as shown in Fig. 7j), and the inhibition of mapk and nfkb signaling pathways by yersinia yopj (as depicted in Fig. 7i) (Fig. 7f–j, Table 5).

**Table 4:** GSEA analysis of dataset GSE22491

Description	setSize	enrichmentScore	NES	p.adjust	qvalue
MEISSNER_BRAIN_HCP_WITH_H3K27ME3	249	0.503121053	2.146625063	0.000687613	0.000578989
REACTOME_REGULATION_OF_BETA_CELL_DEVELOPMENT	41	0.630076836	2.018288026	0.002823127	0.002377152
TIAN_TNF_SIGNALING_VIA_NFKB	24	0.663731652	1.888120045	0.017734735	0.014933142
REACTOME_REGULATION_OF_GENE_EXPRESSION_IN_BETA_CELLS	21	0.659818552	1.817944203	0.036717733	0.030917356
WP_RETINOL_METABOLISM	15	0.714135749	1.81003537	0.038776102	0.03265056
REACTOME_DISEASES_ASSOCIATED_WITH_O_GLYCOSYLATION_OF_PROTEINS	62	0.484733657	1.684690775	0.035294294	0.02971878
PEREZ_TP53_AND_TP63_TARGETS	183	0.402462114	1.653887367	0.003697602	0.003113484
GALINDO_IMMUNE_RESPONSE_TO_ENTEROTOXIN	74	0.454935244	1.63439043	0.043640916	0.036746869
HAMAI_APOPTOSIS_VIA_TRAIL_DN	185	0.382843268	1.575452098	0.013289263	0.011189931
MARZEC_IL2_SIGNALING_UP	109	0.409677998	1.566609626	0.04067462	0.034249165

GSEA: Gene Set Enrichment Analysis。

**Table 5:** GSEA analysis of dataset GSE99039

Description	setSize	enrichmentScore	NES	p.adjust	qvalue
WP_PHOTODYNAMIC_THERAPYINDUCED_NFKB_SURVIVAL_SIGNALING	35	0.707521389	2.183941919	0.001097944	0.000872237
STAMBOLSKY_TARGETS_OF_MUTATED_TP53_DN	49	0.591286098	1.952844229	0.004725774	0.003754287
WP_MAPK_AND_NFKB_SIGNALING_PATHWAYS_INHIBITED_BY_YERSINIA_YOPJ	12	0.797077357	1.920496297	0.013159577	0.010454336
CONCANNON_APOPTOSIS_BY_EPOXOMICIN_UP	223	0.449345412	1.85085634	0.001097944	0.000872237
WP_TNFRELATED_WEAK_INDUCER_OF_APOPTOSIS_TWEAK_SIGNALING_PATHWAY	41	0.57447551	1.832901294	0.016898466	0.013424613
PID_TNF_PATHWAY	44	0.55801217	1.805798904	0.019370979	0.015388846
BIOCARTA_NFKB_PATHWAY	21	0.647200178	1.790176355	0.04823662	0.038320518
WP_APOPTOSIS_MODULATION_AND_SIGNALING	84	0.471195409	1.706221021	0.019943332	0.01584354
WP_BRAINDERIVED_NEUROTROPHIC_FACTOR_BDNF_SIGNALING_PATHWAY	143	0.433280477	1.693557874	0.006699659	0.005322396
WP_TNFALPHA_SIGNALING_PATHWAY	91	0.4570586	1.675224504	0.020792999	0.016518539

GSEA: Gene set enrichment analysis

Additionally, we conducted a Venn diagram analysis to identify the intersection of all significantly enriched functional pathways obtained from the GSEA of the GSE22491 and GSE99039 datasets (Fig. 7K). The findings indicated that a total of 107 functional pathways exhibited significant enrichment in both datasets related to PD. These pathways encompassed targets of nup98 hoxa9 fusion 16d dn, platelet-specific genes, and multiple myeloma pca1 up.

### PPI network of IMRDEGs, mRNA-miRNA, mRNA-TF, and mRNA-drug interaction networks

**Fig. 8:**
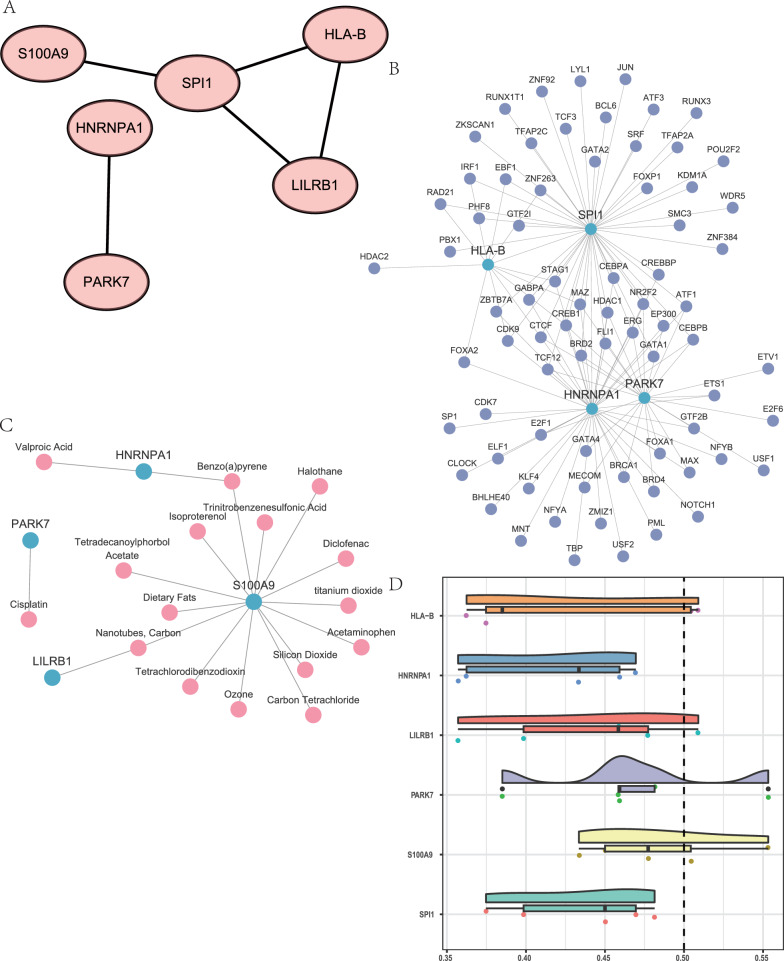
The mRNA-TF (transcription factor), mRNA-protein interaction (PPI), and mRNA-drug interaction networks of the major genes are displayed.IMRDEG PPI network, in (**A**). The mRNA-TF (**B**) and mRNA-drugs (**C**) key gene interaction networks are displayed in (**B**–**C**). The mRNA-TF interaction network (**B**) uses purple circles to symbolize TFs and light blue circles to represent mRNAs. The mRNA-drugs interaction network (**C**) illustrates that mRNAs are shown as light blue circles, while medications are shown as pink circles. **D** Results of functional similarity analysis for important genes are displayed. Transcription factors, or TFs

Using the STRING database, PPI analysis was performed on six IMRDEGs that were differentiable between the disease and control groups and were discovered in the PD dataset. A PPI network with a minimal required interaction score of low confidence (0.150) was constructed by combining these six IMRDEGs, or hub genes. The interaction relationships were shown visually using Cytoscape software (Fig. 8A).

The transcription factors (TFs) that exhibit binding affinity towards the hub genes were identified through a comprehensive search of the CHIPBase and hTFtarget databases (version 2.0). We downloaded the interaction connections from both databases and crossed them with the six hub genes. This process yielded interaction data between four hub genes (*HLA-B, HNRNPA, PARK7, SPI1*) and 74 TFs. These interactions were also visualized using Cytoscape, with sky blue circles representing mRNAs and purple circles representing TFs (Fig. 8B). The hub gene SPI1 had the most significant associations with transcription factors (TFs) within the 46 mRNA-TF interaction pairs observed in the mRNA-TF interaction network. The specific interactions between mRNA and TF are shown in Table S1.

Moreover, potential medications or chemical compounds that target the six hub genes were found using the CTD database. We discovered 16 possible medications or molecular compounds that match the hub genes through CTD. In the mRNA-drug interaction network, medicines are represented by pink circles, and mRNAs are represented by sky-blue circles (Fig. 8C). The *S100A9* gene is targeted by 14 of these drugs or molecular compounds, which is noteworthy. Table S2 provides comprehensive relationships between mRNA and drugs.

Subsequently, an examination was conducted to assess the functional similarities among the six IMRGs. The R package GOSemSim was utilized to assess the semantic similarity among individual GO items, groups of GO terms, gene products, and gene clusters. Next, we showed the results of the functional similarity study between the six *IMRGs* using a cloud-rain plot (Fig. 8D). Out of the six *IMRGs, LILRB1, PARK7, S100A9,* and SPI1 exhibited the highest functional similarity values with other IMRGs, according to the results (Fig. 8D).

### Immune infiltration analysis of PD dataset (CIBERSORT)

**Fig. 9:**
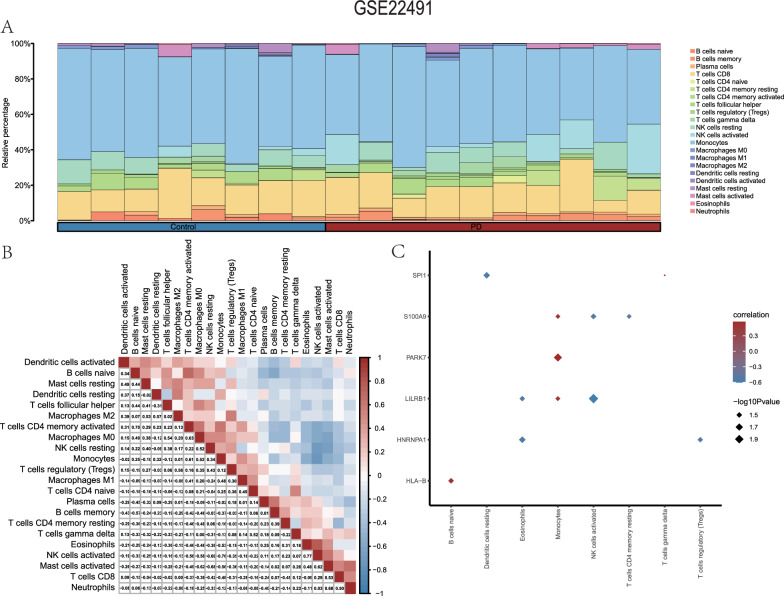
Immune infiltration analysis (CIBERSORT) of the GSE22491 dataset. **A** A bar graph showing the GSE22491 dataset's immune cell infiltration results **B** The findings of the correlation analysis on the GSE22491 dataset indicate the degree of immune cell infiltration. **C** A heatmap from the GSE22491 dataset illustrates the relationship between immune cells and important genes. The correlation heatmap shows a strong relationship when the correlation coefficient's absolute value is over 0.8, a moderate correlation between 0.5 and 0.8, a weak correlation between 0.3 and 0.5, and no linkage below 0.3

The Pearson algorithm and the CIBERSORT package were used to compute the expression profile data from the GSE22491 Parkinson's disease dataset and the association between 22 distinct immune cell types. Based on the findings of the immune infiltration investigation, a bar chart was generated to visually represent the quantity and state of immune cell infiltration for each of the 22 distinct immune cell types inside each sample of the GSE22491 dataset (Fig. 9A).

Next, we determined if the infiltration abundances of the 22 different types of immune cells in the samples from the GSE22491 dataset correlated, and we showed the findings (Fig. 9B). It was observed that there was a negative correlation between the infiltration abundances of the majority of the 22 immune cell types. Interestingly, the biggest negative association (r = − 0.76) was discovered between activated NK cells and monocytes, whereas the strongest positive relationship (r = 0.70) was discovered between T cells with CD4 memory activation and naive B cells.

The simultaneous assessment of the relationship between the expression levels of the six hub genes and the infiltration abundances of the 22 immune cell types in the GSE22491 dataset samples was conducted (Fig. 9C). The study's conclusions showed statistically significant partial correlations (P < 0.05) between the abundances of eight different types of immune cells and the expression levels of the six hub genes in the GSE22491 dataset (Fig. 9C). Notably, B cells naive showed a prominent positive correlation with *HLA-B*, while Monocytes exhibited a significant positive correlation with *LILRB1*, *PARK7*, and *S100A9*. Dendritic cells at rest and SPI1 showed a significant negative correlation; Eosinophils and *HNRNPA1* and *LILRB1* showed a prominent negative correlation; NK cells in activation showed a significant negative correlation with *LILRB1* and *S100A9*; T cells CD4 memory resting and S100A9 showed a notable negative correlation; and T cells regulatory (Tregs) and HNRNPA1 showed a notable negative correlation.

**Fig. 10:**
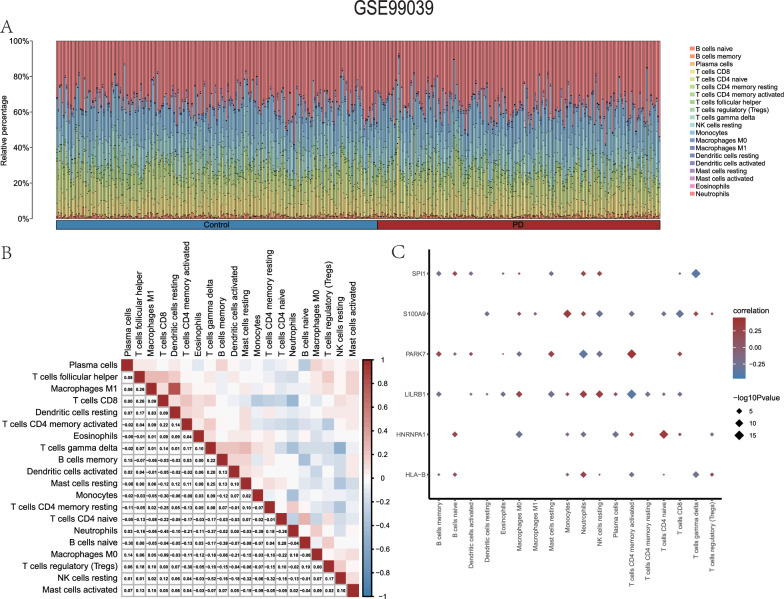
The GSE99039 dataset’s immune infiltration analysis (CIBERSORT). **A** Bar chart of GSE99039 immune cell infiltration results. **B** The results of the correlation analysis on the GSE99039 dataset show how frequently immune cells infiltrate. **C** A heatmap illustrating the relationship between immune cells and significant genes in the GSE99039 dataset. The correlation heatmap gives a vivid picture of the correlation coefficient's intensity. A correlation coefficient larger than 0.8 indicates a high association, while a coefficient between 0.5 and 0.8 suggests a moderate correlation. On the other hand, a result between 0.3 and 0.5 suggests a weak correlation, while anything below 0.3 shows a weak or no association

Next, we carried out the same analysis utilizing the six hub genes found in the GSE99039 dataset. The expression profile data of the Parkinson's disease dataset GSE99039 was found to be correlated with 22 immune cell types using the CIBERSORT tool and the Pearson method. For each of the 20 immune cell types that showed positive values in each sample of the GSE99039 dataset, a bar chart was created to visually represent the infiltration status and abundance. This representation was derived using the results obtained from the immune infiltration study (Fig. 10A).

Afterwards, we determined the relationship between the levels of infiltration of the 20 different types of immune cells in the samples collected from the GSE99039 dataset and displayed the findings (Fig. 10B). According to the findings, the infiltration abundances of the 20 immune cell types exhibited a very balanced range of positive and negative correlations. However, the negative associations were found to be more prominent. The positive correlations among immune cells were all weak; the highest negative correlation (r = − 0.52) was observed between NK cells resting and B cells memory.

The GSE99039 dataset samples’ 20 immune cell types and six hub gene expression levels were determined in the interim (Fig. 10C). The results indicated a significant (P < 0.05) association between the number of immune cell types infiltrating and the expression of the six hub genes in the GSE99039 dataset (Fig. 10C). A notable positive association was found between HLA-B, *LILRB1*, and SPI1 and neutrophils. Furthermore, there was a significant positive association observed between *LILRB1* and *SPI1* and resting NK cells. In addition, it was shown that *PARK7* had a noteworthy favorable association with CD4 memory-activated T cells. In contrast, Neutrophils exhibited a prominent negative correlation with *HNRNPA1* and *PARK7*; T cells CD4 memory activated showed a significant negative correlation with *LILRB1* and *S100A9*; and T cells gamma delta displayed a notable negative correlation with *HLA-B* and *SPI1*.

## Discussion

After a thorough analysis of the IMRDEGs interaction network, we further explored PD-related biomarkers. PD is a chronic neurodegenerative disease whose pathophysiology is very complex and involves interactions between multiple genes and molecules. Therefore, it is particularly important to identify biomarkers that can accurately reflect disease status, predict disease progression, and guide treatment. [19, 20].

In the datasets GSE224913 and GSE99039, we identified six IMRDEGs. The enrichment study primarily focused on the mechanisms of the immune system, including the formation of dendritic cells and the suppression of leukocyte-mediated immunity. Various immune cells were shown to exhibit connections with the six immune-mediated regulatory genes (IMRDEGs). Among the many immune cells examined, neutrophils, resting natural killer (NK) cells, and activated memory CD4 + T cells exhibited the most robust associations. Furthermore, we built regulatory networks for drug interaction that comprise mRNA-targets, mRNA-miRNA, and mRNA-TF, offering insights into putative therapeutic targets and gene regulation processes.

First, we examined IMRDEGs' function in the PD pathophysiology. Numerous studies have demonstrated that the human leukocyte antigen (HLA) has a substantial role in the hereditary susceptibility to PD [21]. The regulation of *HNRNPA* family proteins controls the alternative splicing of CD33, which is associated with Alzheimer's disease [22]. Additionally, significant M2 macrophage infiltration and dysfunctional CD8 + T cell populations are linked to LILRB1 expression, suggesting an immuno suppressive phenotype [23]. Studies show that PARK7 functions as a highly conserved strategy to avert cellular harm from metabolic carbohydrates in Parkinson's disease [24]. Neuroinflammation promotes the diffusion of amyloid protein *S100A9* in the brain tissue, potentially triggering an amyloid cascade reaction involving α-syn and *S100A9*, which leads to PD [25]. The present study investigates the association between the SPI1 haplotype, changed SPI1 gene expression, and the chance of developing Alzheimer’s disease [26]. Changes in the Expression of these genes might directly or indirectly affect neurons’ survival and function, leading to PD’s occurrence and development. Through the construction of an interaction network, a more accessible comprehension of the intricate interconnections among these genes and their potential contributions to the pathological progression of PD was achieved.

In addition, saponins derived from *Plantago asiatica* have the ability to decrease damage to the substantia nigra in rats with Parkinson’s disease. This is achieved by inhibiting HDAC2/MAPK signaling and reducing the polarization of microglia [27]. The implications of their findings suggest that the impairment of the substantia nigra in Parkinson's disease could potentially be mitigated through the inhibition of *HDAC2/MAPK* signaling. The findings of our study suggest that the *HLA-B* marker functions as a downstream factor of *HDAC2* and may impact the substantia nigra injury in Parkinson's disease through HLA-B regulation. Analyzing regulatory modules within the interaction network has revealed clusters of genes that share similar expression patterns and functional annotations. These modules might represent distinct biological processes or pathways perturbed in PD. Through an examination of these modules' roles and interconnections, we may be able to learn more about the immunomodulatory processes that underlie the pathophysiology of Parkinson's disease.

In our study, the use of graphene oxide and KEGG enrichment assays made it possible to systematically evaluate the role of IMRDEGs in various biological processes, molecular activity, cellular components, and important metabolic and signaling pathways. The enrichment analysis provided by these analyses revealed the functional dynamics of imrdeg in the context of disease. The integration of these data highlights the diagnostic and therapeutic relevance of imrdeg, suggesting that their regulation may significantly influence disease outcomes. Future research could build on these insights to better understand the mechanistic role of these genes and provide targeted treatments. In addition, we applied GSEA and GSVA to elucidate the expression patterns of imrdeg in samples with different phenotypic correlations. These analyses identified specific gene sets and pathways significantly associated with these genes, highlighting their roles in distinct biological processes and disease states. Results from GSEA and GSVA demonstrated significant enrichment of IMRDEGs in pathways critical for immune regulation and pathophysiological development, suggesting their potential utility as biomarkers for disease diagnosis. Particularly, their active involvement in inflammatory and immune surveillance pathways underscores their diagnostic relevance, supporting their use in developing diagnostic tools for early disease detection [28]. Overall, integrating these enrichment analysis results provides a comprehensive assessment of the potential diagnostic value of IMRDEGs, enhancing our understanding of their biological functions and offering insights into their application in clinical diagnostics. These findings paved the way for future diagnostic strategies and therapeutic interventions for precision medicine.

Our research assessed the variability in immune cell infiltration levels across datasets containing IMRDEGs. Significant variations in the infiltration patterns of immune cells, including neutrophils, resting NK cells, and CD4 memory-activated T cells, were seen across different datasets. These findings suggest that each dataset reflects a unique immune response associated with a particular disease or environment. Understanding the pathophysiological mechanisms underlying these differences may be crucial. The next analysis revealed robust correlations between many immune cell types, specifically between memory B cells and resting NK cells, indicating a possible synergistic role for these cell types in facilitating immune responses associated with IMRDEGs. Integrating these findings, we discussed how IMRDEGs might influence the Expression of pivotal genes within these key immune cell types. For instance, the upregulation of specific IMRDEGs enhanced JAK-STAT1 pathways in memory B cells, potentially amplifying inflammatory responses [29]. This suggests a direct mechanistic link between IMRDEGs and the modulation of immune responses, which could be critical for developing targeted therapeutic interventions. Our comprehension of immune cell dynamics in disease situations is enhanced by these discoveries, which also highlight the significance of focusing on particular cell types and pathways in order to successfully modify disease outcomes.

In addition, there are some limitations to the current study that must be acknowledged. The analysis was based on bioinformatics data, and thus, the results need to be corroborated by experimental studies. Furthermore, the interaction network was constructed using available data, which might not capture all possible interactions and regulatory mechanisms. To strengthen the accuracy and reliability of the analysis, future studies should strive to include other data sources and experimental validations.

Due to financial reasons, we are unable to carry out experimental verification. We will further demonstrate the modified research in future studies, and we will continue to study in this field in the future.

In conclusion, the six core genes (*HLA-B, HNRNPA, LILRB1, PARK7, S100A9, and SPI1*) play pivotal roles in the progression of PD. The interaction networks encompassing mRNA-TF and mRNA-Drugs contribute significantly to our understanding of disease progression and the optimization of treatment strategies.

## Supplementary Information

Supplementary Material 1.

Supplementary Material 2.

## Data Availability

No datasets were generated or analysed during the current study.
