# Supplementary material for: A comprehensive analysis of the interaction network of immunomodulatory-related differentially expressed genes, aiming to identify biomarkers associated with Parkinson’s disease
Source: Hum Genomics. 2026 Mar 12;20:73. doi: 10.1186/s40246-026-00929-8 (PMC13094144; doi:10.1186/s40246-026-00929-8)
Supplement: Supplementary file 1 — Supplementary Material 1. [file 40246_2026_929_MOESM1_ESM.docx]

**Table S1. mRNA-TF interaction network nodes.**

| mRNA | TF | mRNA | TF |
| --- | --- | --- | --- |
| HLA-B | CDK9 | PARK7 | FLI1 |
| HLA-B | CREB1 | PARK7 | FOXA1 |
| HLA-B | CTCF | PARK7 | GATA4 |
| HLA-B | EBF1 | PARK7 | GTF2B |
| HLA-B | FOXA2 | PARK7 | ATF1 |
| HLA-B | HDAC2 | PARK7 | HDAC1 |
| HLA-B | IRF1 | PARK7 | MAX |
| HLA-B | MAZ | PARK7 | MAZ |
| HLA-B | RAD21 | PARK7 | MECOM |
| HLA-B | SPI1 | PARK7 | NR2F2 |
| HLA-B | STAG1 | PARK7 | BRCA1 |
| HLA-B | ZNF263 | PARK7 | BRD2 |
| HNRNPA1 | ATF1 | PARK7 | BRD4 |
| HNRNPA1 | BHLHE40 | PARK7 | TCF12 |
| HNRNPA1 | BRCA1 | PARK7 | USF1 |
| HNRNPA1 | BRD2 | PARK7 | CEBPB |
| HNRNPA1 | BRD4 | SPI1 | ATF1 |
| HNRNPA1 | CDK7 | SPI1 | ATF3 |
| HNRNPA1 | CDK9 | SPI1 | BCL6 |
| HNRNPA1 | CEBPA | SPI1 | BRD2 |
| HNRNPA1 | CEBPB | SPI1 | CDK9 |
| HNRNPA1 | CLOCK | SPI1 | CEBPA |
| HNRNPA1 | CREB1 | SPI1 | CEBPB |
| HNRNPA1 | CREBBP | SPI1 | CREB1 |
| HNRNPA1 | CTCF | SPI1 | CREBBP |
| HNRNPA1 | E2F1 | SPI1 | CTCF |
| HNRNPA1 | ELF1 | SPI1 | EBF1 |
| HNRNPA1 | EP300 | SPI1 | EP300 |
| HNRNPA1 | ERG | SPI1 | ERG |
| HNRNPA1 | ETS1 | SPI1 | FLI1 |
| HNRNPA1 | FLI1 | SPI1 | FOXP1 |
| HNRNPA1 | FOXA1 | SPI1 | GABPA |
| HNRNPA1 | FOXA2 | SPI1 | GATA1 |
| HNRNPA1 | GABPA | SPI1 | GATA2 |
| HNRNPA1 | GATA1 | SPI1 | GTF2I |
| HNRNPA1 | GTF2B | SPI1 | HDAC1 |
| HNRNPA1 | HDAC1 | SPI1 | IRF1 |
| HNRNPA1 | KLF4 | SPI1 | JUN |
| HNRNPA1 | MAX | SPI1 | KDM1A |
| HNRNPA1 | MAZ | SPI1 | LYL1 |
| HNRNPA1 | MNT | SPI1 | MAZ |
| HNRNPA1 | NFYA | SPI1 | NR2F2 |
| HNRNPA1 | NFYB | SPI1 | PBX1 |
| HNRNPA1 | NOTCH1 | SPI1 | PHF8 |
| HNRNPA1 | NR2F2 | SPI1 | POU2F2 |
| HNRNPA1 | PML | SPI1 | RAD21 |
| HNRNPA1 | SP1 | SPI1 | RUNX1T1 |
| HNRNPA1 | SPI1 | SPI1 | RUNX3 |
| HNRNPA1 | STAG1 | SPI1 | SMC3 |
| HNRNPA1 | TBP | SPI1 | SPI1 |
| HNRNPA1 | TCF12 | SPI1 | SRF |
| HNRNPA1 | USF2 | SPI1 | STAG1 |
| HNRNPA1 | ZBTB7A | SPI1 | TCF12 |
| HNRNPA1 | ZMIZ1 | SPI1 | TCF3 |
| PARK7 | CREB1 | SPI1 | TFAP2A |
| PARK7 | CTCF | SPI1 | TFAP2C |
| PARK7 | E2F1 | SPI1 | WDR5 |
| PARK7 | E2F6 | SPI1 | ZBTB7A |
| PARK7 | EP300 | SPI1 | ZKSCAN1 |
| PARK7 | ERG | SPI1 | ZNF263 |
| PARK7 | ETS1 | SPI1 | ZNF384 |
| PARK7 | ETV1 | SPI1 | ZNF92 |

“mRNA”and“TF”represent node；“-”represent edge；TF：Transcription factors.
