# Supplementary material for: A comprehensive analysis of the interaction network of immunomodulatory-related differentially expressed genes, aiming to identify biomarkers associated with Parkinson’s disease
Source: Hum Genomics. 2026 Mar 12;20:73. doi: 10.1186/s40246-026-00929-8 (PMC13094144; doi:10.1186/s40246-026-00929-8)
Supplement: Supplementary file 2 — Supplementary Material 2. [file 40246_2026_929_MOESM2_ESM.docx]

**Table S2. mRNA-drugs interaction network nodes.**

| mRNA | drug | mRNA | drug |
| --- | --- | --- | --- |
| S100A9 | Tetrachlorodibenzodioxin | S100A9 | Dietary Fats |
| S100A9 | Benzo(a)pyrene | S100A9 | Halothane |
| S100A9 | Diclofenac | S100A9 | Isoproterenol |
| HNRNPA1 | Benzo(a)pyrene | S100A9 | Nanotubes, Carbon |
| HNRNPA1 | Valproic Acid | S100A9 | Ozone |
| LILRB1 | Nanotubes, Carbon | S100A9 | Silicon Dioxide |
| PARK7 | Cisplatin | S100A9 | Tetradecanoylphorbol Acetate |
| S100A9 | Acetaminophen | S100A9 | titanium dioxide |
| S100A9 | Carbon Tetrachloride | S100A9 | Trinitrobenzenesulfonic Acid |

“mRNA”and“drug”represent node；“-”represent edge
